# Global Inequality and Injustice in Mortality Burden from Floods and Landscape Fire-Sourced Air Pollution

**DOI:** 10.34133/hds.0417

**Published:** 2026-07-03

**Authors:** Bingjie Zhou, Rongbin Xu, Zhengyu Yang, Yuming Guo

**Affiliations:** ^1^ Chongqing Emergency Medical Center, Chongqing University Central Hospital, School of Medicine, Chongqing University, Chongqing 400044, China.; ^2^ Climate and Air Quality Research Unit, School of Public Health and Preventive Medicine, Monash University, Melbourne, VIC 3004, Australia.

## Abstract

**Background:** Climate change amplifies the frequency and severity of landscape fire and floods, creating disproportionate health risks across countries. Yet, global disparities and injustices in mortality attributable to these climate-related risks remain poorly quantified. **Methods:** We constructed a country-level panel dataset (2000 to 2019) integrating mortality attributable to landscape fire-sourced (LFS) air pollution and floods, national Human Development Index (HDI), and per-capita CO_2_ emissions. Relative inequality was assessed using the Gini and concentration indices, absolute inequality using HDI-based slope estimates, and climate injustice using the slope of attributable mortality rates against per-capita CO_2_ emissions. **Results:** Mortality burdens from both risks were highly unequal across countries. LFS air pollution showed lower relative inequality (Gini = 0.59 for LFS air pollution and 0.82 for floods) but a steeper socioeconomic gradient (concentration index = −0.41 vs −0.28) and a larger absolute slope with HDI. Inequality from LFS air pollution declined modestly over time, whereas flood-related disparities remained less pronounced. Mortality was inversely associated with per-capita CO_2_ emissions, indicating stronger and more persistent climate injustice for LFS air pollution than for floods. **Conclusion:** This study bridges data-driven analysis and policy perspectives to quantify global inequality and injustice in climate-related mortality burdens due to LFS air pollution and floods. This provides essential data to align emission mitigation and adaptive health policies toward greater environmental equity and justice under climate change.

## Introduction

Climate change is intensifying the frequency, duration, and severity of extreme weather events, creating escalating threats to human health worldwide. Rising global temperatures, altered precipitation patterns, and increasing atmospheric moisture have collectively fueled more frequent and intense wildfires, floods, droughts, and heat waves, which are now strongly linked to human-induced climate change [1–3]. Systematic reviews and epidemiological studies demonstrate that exposure to wildfire-sourced fine particulate matter (PM_2.5_) markedly increases all-cause mortality and respiratory and cardiovascular morbidity, with consistent evidence across diverse populations and geographic settings [4–6]. Meanwhile, flood events are consistently associated with increased rates of infectious disease, hospital admissions, and mortality worldwide [7–10]. Recent evidence has quantified the health impacts on global morbidity and mortality, establishing them as a measurable component of the global burden of disease [2,11,12]. For instance, an estimated 1.5 million deaths per year globally were attributable to landscape fire-sourced (LFS) air pollution [5], and flood exposure accounts for at least 33,000 under-5 deaths per year across 100 low- and middle-income countries (LMICs) [13]. Importantly, these risks are projected to intensify under continued climate change. Anthropogenic warming has already substantially increased wildfire activity globally [3,14], and wildfire-related premature mortality is projected to increase approximately 6-fold globally by the end of the century [15]. Global flood exposure is similarly projected to rise from 1.6 to 1.9 billion people by 2100, with flood frequency expected to increase in many regions as warming continues [16].

Yet, the health burden from these hazards is far from evenly distributed. A growing body of evidence demonstrates that the impacts of climate-related disasters disproportionately affect LMICs, marginalized populations, and regions with limited adaptive capacity [17–19], reflecting a pattern of global inequality where countries with the least resources consistently bear the highest burdens. Specifically, flood mortality rates are substantially higher in low-income and highly unequal societies, where infrastructure deficits and limited emergency response capacity amplify vulnerability [20,21]. Meanwhile, LFS air pollution constitutes a relatively chronic and transboundary hazard, with health impacts amplified by inequalities in air quality monitoring and pollution exposure [22] and inadequate healthcare system capacity typical of LMICs [23]. Beyond unequal distribution, recent analyses have further underscored the concept of climate injustice, the misalignment between countries’ contributions to climate change and the health consequences they bear [24,25]. Per-capita CO_2_ emissions serve as the preferred metric of this misalignment because they represent each nation’s contribution to anthropogenic environmental burden through industrialization and fossil fuel consumption and are the most widely used and theoretically grounded indicator of national climate responsibility in the justice literature [24–26]. The wealthiest top 10% of individuals are responsible for 48% of global greenhouse gas emissions, while the bottom 50% contribute only 12% [27], with this wealthiest 10% having caused two-thirds of global warming and up to 7 times the average contribution to heat extremes [28]. Yet low-Human Development Index (low-HDI) countries bear higher climate-attributable health burdens while facing the least insurance coverage for climate-related losses [1,29]. These patterns underscore that climate injustice is not merely an abstract ethical concern but a measurable and mounting public health reality. Simultaneous quantification of both dimensions is essential, as distributional inequality in health impact and emissions-based injustice do not necessarily converge across countries.

Despite increasing recognition of climate-related inequality, systematic quantification of global disparities in climate-attributable mortality remains insufficiently characterized. Previous studies have laid a methodological foundation by applying inequality metrics such as the Gini index and slope index of inequality to the Global Burden of Disease dataset to track overall health disparities [21,30]. More recent research has extended these approaches to environmental health, documenting that global PM_2.5_ exposure inequality has been high and rising, with the Gini index increasing from 0.30 in 2000 to 0.35 in 2020 [31], and that mortality attributable to ambient air pollution is even more unequally distributed than exposure itself [22]. Global socioeconomic disparities in LFS air pollution exposure have similarly been established, with concentrations more than 4 times higher in low-income countries than in high-income countries [32]. For floods, within-country income inequality has been identified as a key predictor of flood mortality [20]. While these studies provide valuable insights, they largely focus on exposure or within-country income disparities, and critically, neither hazard has been examined within a framework that simultaneously integrates development-level indicators and per-capita CO_2_ emissions to capture both inequality and injustice dimensions.

A comprehensive understanding of climate-related health inequality requires examining both chronic, diffuse exposures and acute, high-intensity climate shocks. Wildfires create sustained and widespread smoke exposure, whereas floods trigger abrupt, localized water-related disasters. Together, they span a broad spectrum of climate–health mechanisms and thus offer a more complete lens for evaluating global disparities. However, no study has jointly assessed these 2 hazards within a unified global inequality–injustice framework. To fill these gaps, this study conducted a global assessment of inequality and injustice in mortality attributable to LFS air pollution and floods, compiling multi-year, country-level datasets on hazard-specific mortality and socioeconomic indicators to quantify disparities across nations and evaluate the degree to which mortality burdens from climate-related disasters are misaligned with nations’ responsibility for climate change.

## Methods

### Data source

We constructed a country-level panel dataset covering the years 2000 to 2019 by compiling multiple global databases on health outcomes, environmental exposure, and socioeconomic development indicators. Estimates of all-cause mortality rates attributable to LFS air pollution were derived from our previously published global health impact assessment [5]. Flood-related mortality was obtained from the Emergency Events Database (EM-DAT), which provided official total death counts for reported flood events worldwide. The database records “total deaths” as the sum of confirmed fatalities directly imputed to the disaster and persons reported missing whose whereabouts since the disaster are unknown and who are presumed dead based on official figures. For each country and year, the flood-attributable mortality rate was calculated as the total number of deaths from all flood events divided by the corresponding midyear population. Annual country-level population estimates were obtained from the United Nations World Population Prospects [33]. Country-level carbon dioxide (CO_2_) emissions, expressed in metric tons of CO_2_ equivalent per capita and excluding land use, land-use change, and forestry, were extracted from the World Development Indicators database of the World Bank [34]. The HDI, reflecting composite achievements in health, education, and income, was obtained from the United Nations Development Programme (UNDP). HDI was selected as the primary socioeconomic indicator because it captures multidimensional development, encompassing health, education, and living standards, rather than economic output alone, making it a more comprehensive proxy for countries’ structural capacity to prevent and respond to climate-related health risks, consistent with previous global health inequality analysis based on Global Burden of Disease data [30,35,36] and recent Lancet Countdown reports [1,19]. Countries were classified into 4 HDI groups following the UNDP categories and into 4 CO_2_ emission groups based on quartiles of per-capita emissions, with both variables labeled as low, medium, high, and very high.

### Data analysis

The analysis covered the period from 2000 to 2019 and included country-level data from 201 countries for LFS air pollution and 178 countries for floods. The key variables analyzed included annual attributable mortality rates (AMRs) to LFS air pollution and floods, HDI, and per-capita CO_2_ emissions. HDI was used to represent cross-country socioeconomic development, while per-capita CO_2_ emissions served as an indicator of national contributions to anthropogenic climate change.

To quantify cross-national inequalities in environmental-hazard-attributable mortality, we used both relative and absolute measures. Relative inequality was assessed using the Gini index and concentration index. The Gini index was derived from the Lorenz curve, based on countries ranked by their mortality rates, whereas the concentration index was derived from a concentration curve based on countries ranked by their HDI levels. The Gini index captures overall inequality in mortality distribution, while the concentration index reflects mortality inequality associated with socioeconomic development in this study. The Gini index ranges from 0 (perfect equality) to 1 (maximum inequality), whereas the concentration index ranges from −1 to +1, with 0 indicating perfect equality, negative values indicating greater mortality in lower-HDI countries, and positive values indicating the opposite. All indices were computed at the country level without population weighting (i.e., each country was treated as equal), as the focus was on inequality between countries rather than within-country population disparities and to avoid large countries (e.g., China and India) disproportionately influencing global estimates. Absolute inequality was estimated using linear regression, expressed as the change in AMR per 0.1-unit increase in HDI. This slope-based measure reflects the absolute magnitude of mortality differences across the HDI gradient, complementing relative indices that describe proportional inequality.

To examine the patterns of climate injustice, defined as the degree of misalignment between countries’ contributions to climate change and their mortality burdens, we quantified this relationship using linear regression between AMR and per-capita CO_2_ emissions. The regression coefficient was expressed as the change in AMR per 10% increase in log-transformed per-capita CO_2_ emissions. Log transformation was applied to reduce the skewness of the CO_2_ distribution and to allow interpretation as proportional changes in mortality.

All analyses were conducted at the country level for each year, and results were reported separately for LFS air pollution- and flood-attributable mortality. All statistical analyses and visualizations were performed in R (version 4.5.1). All visualizations used ggplot2 (version 3.5.2) as the foundation. Geographic maps were additionally constructed using the sf package (version 1.0.21) with shapefiles from Natural Earth (1:110 m scale) and colored with the viridis scale (version 0.6.5). Lorenz curves and Gini indices were calculated using the ineq package (version 0.2.13). Multipanel figures were assembled using cowplot (version 1.2.0) and egg (version 0.4.5).

## Results

**Fig. 1.**
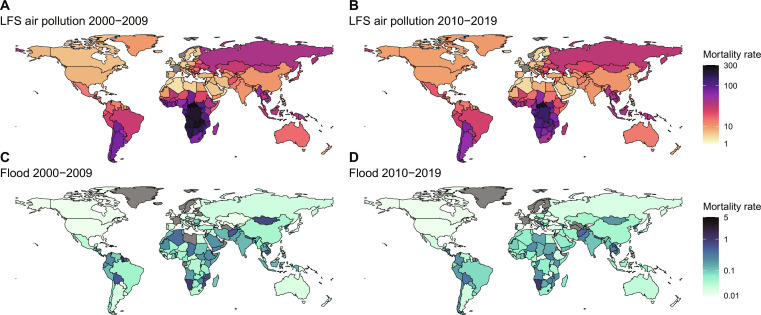
Global distribution of mortality rates from landscape fire-sourced (LFS) air pollution and floods. Panels (A) and (B) show the average annual mortality rate (per 100,000 persons) from LFS air pollution during 2000 to 2009 and 2010 to 2019, respectively. Panels (C) and (D) present the corresponding mortality rates associated with flood events for the same periods. Mortality rates are displayed on a logarithmic scale to improve cross-regional comparability. Darker shades indicate higher mortality rates, highlighting spatial disparities and temporal trends across regions.

Globally, mortality attributable to LFS air pollution and floods exhibited pronounced spatial heterogeneity (Fig. 1). The highest mortality rates for LFS air pollution were concentrated in southern South America, sub-Saharan Africa, and Southeast Asia, while most high-income regions showed comparatively low burdens. Flood-related mortality was particularly elevated in northern South America, parts of Africa, eastern Mediterranean areas, and Southeast Asia. Spatial contrasts in both mortality burdens persisted between 2000–2009 and 2010–2019, although overall intensities appeared diminished in the latter decade, most notably across Africa.

**Fig. 2.**
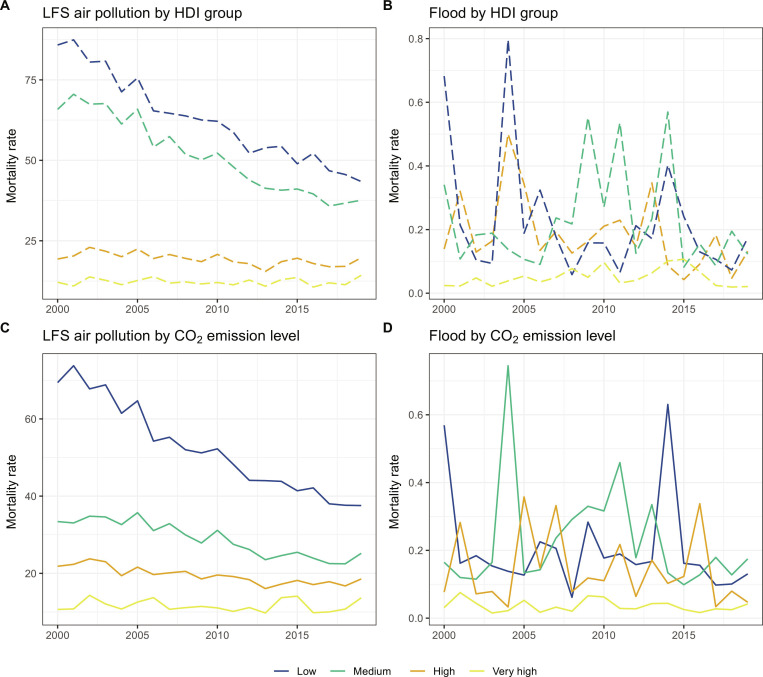
Global trends of mortality rates attributable to landscape fire-sourced (LFS) air pollution and floods by socioeconomic and emission groups, 2000 to 2019. Panels (A) and (B) show annual mortality rates associated with LFS air pollution and floods, respectively, across countries worldwide, stratified by Human Development Index (HDI) levels. Panels (C) and (D) display corresponding trends stratified by CO_2_ emission levels.

When countries were grouped by HDI and CO_2_ emission level, distinct gradients in mortality burden were observed (Fig. 2). Mortality attributable to LFS air pollution showed clear separation across both HDI and CO_2_ emission groups, indicating systematic differences linked to socioeconomic development and emission responsibility. Countries with low and medium HDI or low CO_2_ emissions experienced the highest mortality rates, suggesting disproportionate health impacts in less developed and low-emission nations. Although these groups showed a modest declining trend over time, their mortality rates remained consistently higher than those in more advantaged groups. Meanwhile, higher-HDI and higher-emission groups maintained low and stable mortality levels, indicating potential advantages in adaptive capacity and resilience. For flood-related mortality, group differences were less consistent, and no clear gradient was evident across most categories. However, the very high-HDI and very high-emission groups consistently showed minimal mortality, indicating relative insulation from flood impacts compared with lower-HDI and low-emission groups.

**Fig. 3.**
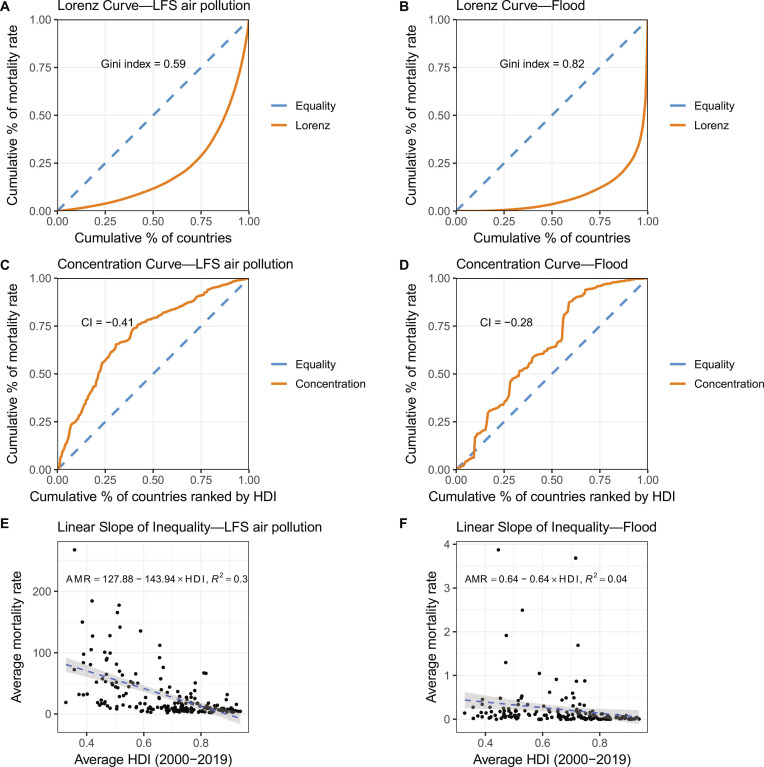
Global inequality in mortality from landscape fire-sourced air pollution and floods, 2000 to 2019. Panels (A) and (B) show Lorenz curves illustrating unequal global distributions of mortality. Panels (C) and (D) present concentration curves ranked by Human Development Index (HDI), capturing relative inequality. Panels (E) and (F) display linear slopes of inequality, capturing the absolute inequality of average mortality rate against average HDI.

Quantitative inequality metrics confirmed substantial cross-country differences in mortality burdens (Fig. 3). For relative inequality, the Gini index was 0.59 for LFS air pollution and 0.82 for floods, indicating high global inequality in both hazards, with flood-related mortality showing a greater disparity among countries. The corresponding Lorenz curves deviated markedly from the line of equality, illustrating that a small proportion of countries accounted for a disproportionately large share of global deaths. Global inequalities were further illustrated by the concentration curves ranked by HDI, which yielded negative concentration indices (−0.41 for LFS air pollution and −0.28 for floods). These negative values indicate that mortality was disproportionately concentrated among less developed countries, with a steeper socioeconomic gradient for LFS air pollution than for floods. Linear slopes of inequality between average mortality and HDI captured absolute inequality, further reinforcing these inverse associations. For LFS air pollution, the regression slope (*β* = −143.94) corresponded to a 14.4-unit decrease in AMR (deaths per 100,000 persons) for every 0.1-unit increase in HDI (*R*^2^ = 0.32), whereas for floods, *β* = −0.64 indicated only a 0.064-unit decrease for the same change in HDI (*R*^2^ = 0.04).

**Fig. 4.**
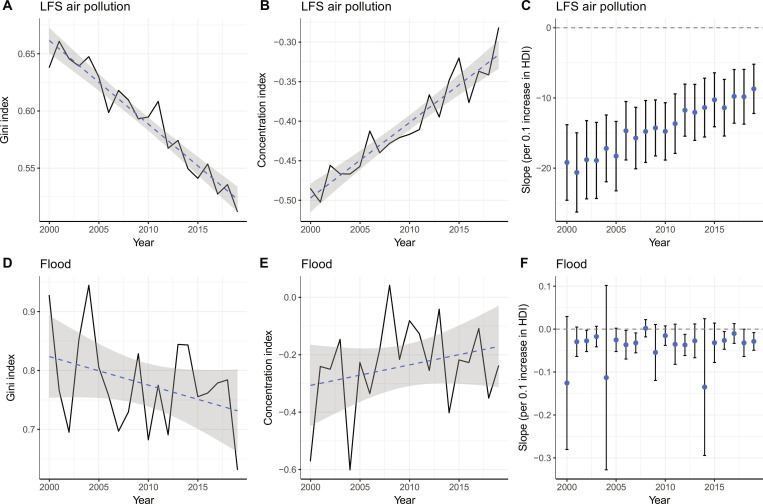
Temporal trends in global country-level inequality in mortality from landscape fire-sourced (LFS) air pollution and floods, 2000 to 2019. Panels (A) to (C) show the temporal changes in annual Gini index, concentration index, and slope of socioeconomic inequality (change in mortality rate per 0.1 increase in Human Development Index [HDI]) for LFS air pollution from 2000 to 2019. Error bars represent the confidence intervals of the estimated slopes. Panels (D) to (F) show the corresponding indices for flood-attributable mortality.

We further examined the temporal trends of inequality indices from 2000 to 2019 (Fig. 4). For LFS air pollution, the Gini index declined gradually toward zero, while both the concentration index and the slope of inequality increased from strongly negative values toward neutrality. These trends indicate a progressive reduction in inequality over time, reflecting modest improvements in both relative and absolute dimensions. In contrast, the inequality indices for flood-related mortality exhibited greater fluctuations. Although the overall direction of change was similar, the decline in the Gini and concentration indices was less consistent and less pronounced than that observed for LFS air pollution, and the slope of inequality remained largely stable over time, suggesting weaker or stagnant progress toward equality in flood mortality.

**Fig. 5.**
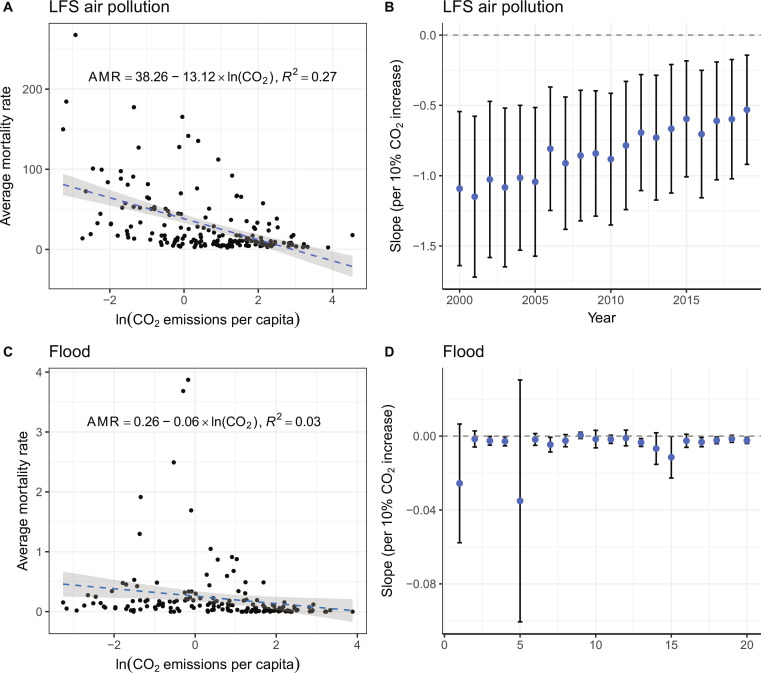
Global injustice patterns in mortality from landscape fire-sourced (LFS) air pollution and floods, 2000 to 2019. Panels (A) and (B) show the relationships between average mortality rate and log-transformed CO_2_ emissions per capita and the temporal trends in the slope of injustice (per 10% increase in CO_2_ emissions) for LFS air pollution. Panels (C) and (D) present the corresponding results for flood-related mortality. Error bars represent the confidence intervals of the estimated slopes.

Lastly, the association between mortality rate and CO_2_ emissions further illustrated the global patterns of climate injustice (Fig. 5). Both the temporal trends and the strength of these associations closely mirrored the inequality patterns observed with HDI. Mortality burdens were inversely associated with CO_2_ emissions, indicating that countries contributing least to global emissions experienced the highest health losses. For LFS air pollution, the regression coefficient (*β* = −13.12, *R*^2^ = 0.27) corresponded to a −1.25-unit change in AMR (deaths per 100,000 persons) per 10% increase in CO_2_ emissions, whereas the association for floods was much weaker (*β* = −0.06, *R*^2^ = 0.03), equivalent to a −0.006-unit change in AMR per 10% increase. When comparing the 2 climate-related risks, LFS air pollution showed a stronger but gradually improving association with national CO_2_ emissions, whereas flood mortality showed no consistent improvement over time. Overall, the manifestation of climate injustice was less evident for floods than for LFS air pollution, as reflected by its smaller slope and the absence of a clear temporal trend.

## Discussion

This study provides the first quantitative global assessment of inequality and injustice in mortality attributable to LFS air pollution and floods. Both climate-related risks exhibit pronounced relative and absolute inequality, with mortality burdens disproportionately concentrated in LMICs. Although the global inequality of these mortality burdens has gradually declined over time, substantial gaps remain. Beyond inequality, our analysis quantified the dimension of climate injustice, revealing a misalignment between climate responsibility and mortality burdens, especially mortality attributable to LFS air pollution. This injustice underscores how countries with minimal emissions continue to bear disproportionate mortality burdens linked to global climate change.

The stronger socioeconomic gradient but weaker relative disparity of mortality burden observed for LFS air pollution compared with floods reflects the distinct nature of these 2 climate-related risks. LFS air pollution represents a chronic, spatially diffuse and transboundary risk that accumulates through recurrent fire seasons and transboundary smoke transport [37], amplifying long-term cardiopulmonary mortality [38]. Because exposure to LFS air pollution extends across wide regions and prolonged periods, its cumulative health impacts are strongly shaped by structural differences in air quality, healthcare access, and adaptive capacity [23] that parallel national development [39]. Such widespread exposure lowers relative inequality, as most countries are affected, but intensifies the socioeconomic gradient, as these structural disparities dictate the mortality burden. In contrast, floods constitute acute and highly localized shocks driven by extreme precipitation and land-use change. Their mortality risk depends on infrastructure robustness, emergency preparedness, and governance capacity [40,41], which also tend to vary by socioeconomic level. Consequently, flood mortality still reflects pronounced inequalities, particularly where poverty, informal settlements, and inadequate disaster management intersect [20,42]. However, because these impacts are episodic and concentrated in a few disaster-prone hotspots, they manifest as high relative disparity and are less linearly tied to national development status than that of LFS air pollution across the global spectrum.

Beyond documenting the unequal distribution of mortality across countries’ development spectrum, our analysis quantifies an additional ethical dimension: the degree of mismatch between climate responsibility and health consequences. Correlating attributable mortality with per-capita CO_2_ emissions is the preferred approach to quantifying this dimension because it simultaneously captures both quantities that define climate injustice: each nation’s contribution to the anthropogenic climate burden and each nation’s health consequences from fire and flood hazards. The slope between them provides a direct empirical measure of their misalignment, operationalizing climate injustice as conceptualized in the justice literature [24,43]. Furthermore, the emissions–mortality association we observed served as a conservative proxy for climate injustice as it captures more than the climate-attributable fraction of fire and flood mortality. While anthropogenic warming is a documented global driver of fire weather and flood risk [44–46], the resulting mortality is intrinsically linked to nonclimatic factors, such as land-use change, informal urban expansion, and deficits in emergency management [23,39,47]. Crucially, these factors are systematically concentrated in low-emission nations as a consequence of their specific historical industrial trajectories [25,48], functioning as “costressors” that amplify mortality with climate factors along the same global emissions axis.

Compounding this injustice, the disproportionate increases in fire weather and flood risk concentrate in tropical and monsoon-affected regions that overlap closely with countries in the lowest tiers of per-capita emissions [49,50]. Even where high-emission countries experience climate-amplified hazards, robust infrastructure and emergency systems translate exposure into far fewer deaths [19,51]. The unequal distribution of climate effects does not dilute the injustice signal but constitutes its physical substrate. The negative slope quantifies an aggregate climate injustice signal: populations contributing least to anthropogenic warming bear the largest share of these mortality burdens, whether driven by climate amplification, capacity deficits, or their synergistic interaction. Notably, the stronger injustice signal for LFS air pollution compared to floods reflects its chronic, transboundary nature, which allows capacity gaps to compound across seasons, creating a more persistent misalignment between emissions responsibility and health impacts than episodic flood events.

Our findings are broadly consistent with previous research documenting unequal global distributions of climate-related health burdens [20,22,25,40,42]. We observed that mortality from LFS air pollution and floods remains disproportionately concentrated in lower-income and low-emission countries. However, this study extends prior evidence in several important ways. First, it provides the first global assessment to directly compare chronic LFS air pollution and acute flood risks within a unified analytic framework, allowing systematic examination of how different types of climate hazards generate distinct inequality and injustice patterns. Second, it introduces a dual-dimensional approach that quantifies both inequality through Gini and concentration indices and injustice through CO_2_–mortality slope analyses, transforming normative concepts of fairness and responsibility into empirical metrics. Third, it enhances the policy and ethical interpretability of quantitative findings by linking inequality metrics with questions of accountability and justice in climate and health governance. Together, these advances illuminate how economic development, emissions responsibility, and hazard exposure jointly determine health vulnerability to climate change.

By integrating standardized inequality and injustice metrics across 2 contrasting climate-related risks, LFS air pollution and floods, we quantified not only the uneven distribution of mortality but also the ethical imbalance between those responsible for emissions and those bearing the health consequences, emphasizing the need to align emission mitigation with equitable adaptation and health protection strategies. Our analytical framework translates abstract principles of fairness and accountability into measurable indicators, thereby operationalizing climate justice and providing a scalable tool for monitoring climate-related health disparities and guiding equitable adaptation strategies. These insights point to concrete actions across governance levels. At the international level, embedding inequality and injustice metrics into accountability frameworks enables mitigation progress to be evaluated in terms of equity rather than emission totals alone, supported by governance mechanisms that link emission accountability to health protection through transparent reporting, cross-sectoral coordination, and enforceable standards. At the national level, mitigation leadership by high-income and high-emitting countries, through strengthened emission reduction commitments, accelerated transitions away from fossil fuels, and alignment of mitigation targets with their transboundary health impacts, is essential to address structural injustice. In parallel, less developed and low-emission countries require priority support for health system strengthening, early-warning systems, and climate-resilient infrastructure, alongside targeted interventions for vulnerable populations and regions where chronic environmental exposures intersect with limited adaptive capacity. Bridging data-driven evidence with ethical and governance considerations will be essential to ensure that progress in climate mitigation and adaptation translates into progress in global health equity.

This analysis has several limitations. The use of country-level aggregation may mask substantial within-country disparities, particularly in large and socioeconomically diverse nations. Furthermore, the ecological nature of the analysis may limit inference at the individual level, as country-level associations do not necessarily reflect within-country relationships. That said, country-level data provide the most consistent and comparable global coverage currently available. Future research should build on this foundation by incorporating subnational data to capture local variability in exposure and vulnerability. In addition, the current framework does not explicitly include indicators of adaptive capacity or policy response, which are essential for interpreting national resilience. While our inequality and injustice indices indirectly reflect such capacity gaps through socioeconomic and emission gradients, future studies should explicitly incorporate adaptation metrics such as early-warning systems, governance indicators, and health infrastructure to better capture the mechanisms underlying observed disparities. Expanding this framework to other hazards, including heat extremes, droughts, or cyclones, will further strengthen the empirical basis for equitable and adaptive climate–health policy. Finally, our flood mortality data from EM-DAT predominantly capture direct fatalities (drowning, traumatic injury, structural collapse, and immediate post-event deaths) and do not systematically include indirect mortality from postflood infectious disease outbreaks, healthcare service disruption, and displacement-related morbidity, which is well-documented to concentrate in LMICs with weaker health and sanitation infrastructure [7,9]. As a result, our analysis likely represents a conservative lower bound of flood inequality and injustice estimates for low-emission countries, as it omits these secondary fatalities that empirical evidence shows are heavily skewed toward resource-poor settings. The comparatively weaker injustice slope observed for floods relative to LFS air pollution thus reflects, in part, this systematic underestimation. We acknowledge, however, that incorporating broader morbidity measures (such as disability-adjusted life years that capture mental health burdens) could yield more nuanced patterns, as flood-related mental health impacts are widely documented globally [52]. Future work integrating linked surveillance of postflood infectious disease, mental health, and healthcare disruption outcomes would refine these quantifications.

## Conclusion

This study bridges data-driven analysis and policy perspectives to reveal how environmental and social inequalities in mortality burden converge to shape health outcomes under climate change. We observed improving yet persistent global inequality and injustice in LFS air pollution and flood-related mortality, suggesting that continued actions are needed, including mitigation leadership by high-income countries, targeted adaptation and health protection for less developed and low-emission countries, and greater international accountability that prioritizes equity in evaluating climate action, to mitigate climate inequality and injustice.

## Ethical Approval

This study used publicly available aggregated country-level data from previously published sources and international databases. No individual-level data or human participants were involved. Therefore, ethical approval was not required.

## Data Availability

The data used in this study are publicly available. Mortality attributable to LFS air pollution was obtained from a previously published global health impact assessment [5] (https://doi.org/10.1016/S0140-6736(24)02251-7). Flood-related mortality data were sourced from EM-DAT (https://www.emdat.be/). Country-level population data were obtained from the United Nations World Population Prospects (https://population.un.org/wpp/). Per-capita CO_2_ emissions were extracted from the World Bank World Development Indicators (https://data360.worldbank.org). The HDI was obtained from the UNDP (https://hdr.undp.org/data-center/human-development-index).
